# A Review of Wearable Solutions for Physiological and Emotional Monitoring for Use by People with Autism Spectrum Disorder and Their Caregivers

**DOI:** 10.3390/s18124271

**Published:** 2018-12-04

**Authors:** Mohammed Taj-Eldin, Christian Ryan, Brendan O’Flynn, Paul Galvin

**Affiliations:** 1 Tyndall National Institute, University College Cork, T12 R5CP Cork, Ireland; brendan.oflynn@tyndall.ie (B.O.); paul.galvin@tyndall.ie (P.G.); 2 School of Applied Psychology, University College Cork, T12 R5CP Cork, Ireland; christian.ryan@ucc.ie

**Keywords:** autism spectrum disorder, challenging behavior, emotional monitoring, physiological monitoring, wearable devices

## Abstract

The goal of real-time feedback on physiological changes, stress monitoring and even emotion detection is becoming a technological reality. People in their daily life experience varying emotional states, some of which are negative and which can lead to decreased attention, decreased productivity and ultimately, reduced quality of life. Therefore, having a solution that continuously monitors the physiological signals of the person and assesses his or her emotional well-being could be a very valuable tool. This paper aims to review existing physiological and motional monitoring devices, highlight their features and compare their sensing capabilities. Such technology would be particularly useful for certain populations who experience rapidly changing emotional states such as people with autism spectrum disorder and people with intellectual disabilities. Wearable sensing devices present a potential solution that can support and complement existing behavioral interventions. This paper presents a review of existing and emerging products in the market. It reviews the literature on state-of-the-art prototypes and analyzes their usefulness, clinical validity, and discusses clinical perspectives. A small number of products offer reliable physiological internal state monitoring and may be suitable for people with Autism Spectrum Disorder (ASD). It is likely that more promising solutions will be available in the near future. Therefore, caregivers should be careful in their selection of devices that meet the care-receiver’s personal needs and have strong research support for reliability and validity.

## 1. Introduction

Modern life is intensely social, both in person and online: people interact with much wider social networks than ever before. For some people, intense interaction can expose the person to extreme emotional states which can be distressing in their own right and have been linked as potential causes of chronic cardiovascular diseases, if not treated.

Therefore, physiological changes and physiological markers of emotional states could be monitoring to keep track of the individual’s distress levels and notify the person of concern if the values of such parameters exceed safe limits. With the explosion of Information and Communication (ICT) technologies and the widespread adoption of consumer electronics devices such as wristbands and smart watches, this presents an opportunity for using these technologies to monitor the health and wellbeing of the user at the same time. 

This paper reviews wearable devices that can monitor physiological signals and markers of emotional states for the general population. Besides this, the present work also reviews the devices that can be adopted or designed for people with Autism Spectrum Disorder (ASD). Specifically, it presents the existing commercial solutions, compares their features and associated sensors, and comments on their usefulness.

The rest of this paper is organized as follows: Section 2 gives an overview of Autism Spectrum Disorder (ASD) and discusses emotional arousal and stressors as potential precursors to challenging behavior. Also, it gives an overview of the physiological biomarkers typically used for emotion monitoring and discusses the unique physiological changes in the ASD population compared to the general population. Section 3 provides a survey of relevant review papers that address physiological and emotional monitoring for various applications. Then, Section 4 explains the methodology followed in searching for target devices and solutions and then reviews and compares those devices and research prototypes. Also, it reviews existing wearable physiological and emotion monitoring products, research prototypes and emerging solutions with particular interest to those which can be adopted or designed for people with ASD with a comparative analysis of the devices and prototypes. Specifically, it highlights the features of the devices together with their advantages and potential limitations. Section 5 presents a discussion on the reviewed devices and how they can help people with ASD in their daily life, monitor their emotional states and how they can assist parents and caregivers in improving existing treatment and intervention programs by integrating wearable assistive technology to complement and optimize their treatment and stress management approaches and it discusses open challenges. Section 6 provides a summary of the work and open challenges for adopting wearable assistive technologies by people with ASD, their caregivers, and health support service providers.

## 2. Physiological Changes in ASD Population, Their Relevance in the Context of Anxiety, Stress, and Challenging Behavior Detection, and the Potential for Wearable Assistive Technology

In this section, we will briefly define ASD, give an overview of the physiological biomarkers that can be used for stress detection, and examine the literature on physiological changes associated with emotional arousal such as anxiety and stress.

### 2.1. Autism, and Prevelance of Anxiety in ASD Population

Autism Spectrum Disorder (ASD) is a neurodevelopmental condition characterized by deficits in reciprocal social interactions and communication skills, accompanied by restrictive and repetitive behaviors. Comorbid psychiatric problems are common among children with an ASD [1]. Anxiety and poor stress management in people with ASD are prevalent comorbid psychiatric problems, with reported prevalence rates varying between 11% and 84% [2]. In particular, it has been reported that more than half of the children with an ASD suffer from at least subclinical levels of anxiety [3]. Some of the most frequently reported anxiety disorders and symptoms seen in children with ASD include generalized anxiety disorder and social phobia [2]. In ASD, anxiety can intensify social interaction difficulties, increase levels of ritualized or repetitive behaviors, and magnify irritability and aggression [4]. Some aspects of challenging behaviors are associated with anxiety and negative emotional states [5]. For instance, a link has been established between challenging behaviors and Generalized Anxiety Disorder in young males with ASD [6]. Challenging behaviors are common in individuals with ASD and the impact of the behavior reduces well-being and quality of life. Individuals with ASD are at higher risk of developing challenging behaviors compared to the general population. Aggression in particular, is associated with negative outcomes for children with ASD and their caregivers, including increased stress levels and decreased quality of life [7].

Monitoring the physiological changes associated with negative emotions in people with ASD can empower caregivers by giving them insights into the internal emotional changes and enabling them to understand what such individuals are experiencing in a real-time fashion. This can be particularly important for children and adults with ASD due to the high comorbidity of Alexithymia, which is a commonly associated difficulty in identifying and describing one’s own emotional state [8]. Increasing caregivers’ awareness of the individual’s emotional state, has the potential to allow the caregiver to take necessary actions to alleviate the symptoms of stress and encourage the individual to utilize better coping strategies to manage stress. 

### 2.2. Physiological Signals for Emotional Monitoring

There are various physiological signals and parameters which are used for physiological and emotional assessment. Each parameter is briefly explained in the following.

#### 2.2.1. Heart Rate, Heart Rate Variability, and Heart Rate Reactivity

Heart rate (HR) is a measure of the number of beats per minute and can be used for monitoring the cardiovascular activity of the heart. However, heart rate gives limited information about the heart activity. A more useful measure is called heart rate variability (HRV) which calculates the time intervals between two consecutive R peaks in the ECG signal, if measured from ECG sensor. The gold standard for measuring and calculating HRV typically used is [9] which is widely recognized and used by physiology researchers and clinicians. However, in the context of this review, as most of the reviewed devices in this paper are for the well-being electronics consumer market, very few devices have been validated against this standard. However, most heart rate validation studies use another commonly used device which is widely used for this parameter validation. For example, Polar H7 and Polar H10 are commonly used for heat rate validation studies.

However, in the recent developments of wearable devices, a new class of device that measure heart rate and heart rate variability has emerged, using a photoplethysmography (PPG) sensor on wristbands. PPG sensor relies on a different principle the measurement of Blood Volume Pulse (BVP) to estimate the heart rate and approximated heart rate variability value. Although this seems to provide fairly accurate heart rate measurements, its heart rate variability recording is not as accurate as an ECG sensor and the decision to trust HRV analysis using PPG depends on the parameter one intends to use [10,11].

Another parameter that is closely related to Heart Rate Variability is the Respiratory Sinus Arrhythmia (RSA). RSA can be defined as the heart rate variability in synchrony with respiration, by which the R-R interval on an ECG signal is reduced during inhalation and increased during exhalation [12]. RSA reacts to stress or other emotional experiences and thus can be used to characterize anxiety disorder, by revealing information pertinent to the emotion regulation process. In addition, RSA changes as a function of stressor. For instance, it has been reported that high resting RSA amplitude is associated with a greater withdrawal of stressors and subsequent recovery could represent a flexible and adaptive physiological response system to a challenge [13]. On the other hand, low resting RSA accompanied by an inadequate reactivity to stress could indicate an inappropriate regulatory mechanism. 

#### 2.2.2. Respiration Rate

Respiration rate (RR) is the total number of breaths, or respiratory cycles, that occur each minute. Respiration rate can change due to illness, stress, etc. The respiratory rate is controlled by the respiratory center located within the medulla oblongata of the brain. It has been shown that the respiration rate is a useful indicator for stress [14].

#### 2.2.3. Electrodermal Activity

Electrodermal activity (EDA) is also called Galvanic Skin Response (GSR) measures the conductance of the skin. The EDA can be measured by using two electrodes on the skin surface when one electrode injects an AC current with small amplitude into the skin and the other is used to calculate the impedance of the skin using Ohm’s Law given a certain voltage. It has been reported that EDA can be a useful indicator of stress [15].

#### 2.2.4. Skin and Body Temperature

Skin temperature (ST) refers to the temperature measured on the surfaced of the human skin. Body temperature refers to the temperature of the core body. Skin temperature is easier to measure as it only requires skin contact and thus can be measured using most wearable devices. Research has shown that including body and peripheral temperature can be useful physiological parameter of stress, although this depends on the location of temperature measurement [12].

#### 2.2.5. Cortisol Level

Cortisol is a hormone that is released by the adrenal gland of the human body when the person is exposed to particular external stimuli that causes stress. Cortisol Level refers to the level of cortisol in the body and can be measured conventionally from Saliva. More recently, there has been works reporting the potential for measuring cortisol level non-invasively using wearable patches attached to the skin [16]. When the person experiences stress, his/her autonomous nervous system orders the glands to release hormones including cortisol to cope with stress [12].

#### 2.2.6. Blood Pressure

Blood Pressure (BP) is the pressure of the blood in the circulatory system, often measured for diagnosis. However, another use of blood pressure can be to monitor changes in the emotional state of the person such as stress. When the person is exposed to a stressful situation, the body produces a surge of hormones which temporarily increase the blood pressure.

It has been found that experiencing prolonged and repeated daily stress contributes to an acute rise in blood pressure [17]. In another study, it has been reported that experiencing a combination of physical and mental stress resulted in significantly higher systolic blood pressure. However, having the mental stress by itself did not have an effect on the systolic blood pressure [18].

#### 2.2.7. Blood Volume Pulse

Blood Volume Pulse (BVP) is measured using a photoplethysmography (PPG) and indicates dynamic changes in blood volume underneath the sensor. The BVP signal indicates relative changes in the vascular bed due to vasodilation or vasoconstriction (increase or decrease in blood flow) as well as to changes in the elasticity of the vascular walls. BVP is commonly used to detect heart rate, heart rate variability, and relative blood volume. In the context of stress detection, research has shown features extracted from blood volume pulse can be used to detect stress [19].

#### 2.2.8. Blood Oxygen Saturation

Blood Oxygen Saturation refers to the extent to which hemoglobin is saturated with oxygen. Hemoglobin is an element in the blood that binds with oxygen to carry it through the bloodstream to the organs, tissues, and cells of the body [20]. Normal oxygen saturation is usually between 96% and 98%. Research has shown that stress alters the oxygen saturation [21] which means that it has potential for stress detection.

#### 2.2.9. Electromyography

Electromyography (EMG) refers to the muscle response or electrical activity in response to a nerve’s stimulation of the muscle [22]. The test is used to help detect neuromuscular abnormalities. In the context of stress detection, the literature has reported that muscle activity of certain body parts such as face, shoulder, and lower back exhibit increased EMG activity during stress [23,24] suggesting the potential for using EMG to detect stress.

#### 2.2.10. Electroencephalogram

Electromyography (EEG) refers to the measurement of voltage fluctuations resulting from ionic current within the neurons of the brain. Clinically, EEG refers to the recording of the spontaneous electrical activity of the brain over a certain period of time. To measure EEG signal, electrodes are typically placed on the skin of the head to make a good contact with the scalp and register the electrical potentials due to neuronal activity [25]. The spectral analysis of EEG can be split into several frequency bands, called waves. Those bands have names: Delta for frequencies less than 4 Hz, Theta for frequencies between 4 and 8 Hz, Alpha for frequencies between 8 and 12 Hz, Beta for frequencies between 13 and 31 Hz). EEG has shown good correlation with mental stress in terms of suppression of alpha waves [26] and improvement of theta waves [27]. 

### 2.3. Physiological Changes Associated with Anxiety, Stress in ASD Population

Children and adolescents with ASD experience high levels of anxiety disorders with an estimate prevalence rate of 40% [28]. This varies across anxiety diagnoses [29], but overall the prevalence of is higher than in the general population [30]. 

People with ASD can exhibit different physiological response patterns compared with typically developing peers. For example, Jansen et al. have shown some evidence of variability in heart rate arousal in response to public speaking stressors [31]. Adults with ASD can exhibit decreased heart rate in response to psychosocial stressor compared to non-ASD adults. Also, it was reported that a group with autism was more likely to have a decrease in salivary cortisol following the stressor [32]. In addition, the autonomic arousal represented by electrodermal activity among children was investigated where higher baseline heart rates in ASD participants was found [33]. 

Heart rate variability, as an important physiological biomarker, provides deep insights for the clarification of the relationship between psychological and physiological processes [34]. People with ASD exhibit heightened cardiovascular arousal even without the apparent presence of a stressor [35].

Other researchers have investigated other biomarkers. For instance, Kushki et al. have used Respiratory Sinus Arrhythmia together with heart rate to characterize the cardiac autonomic profile of children with ASD during anxiety [36]. They found that ASD group had an elevated basal heart rate, and showed decreased heart rate reactivity to social anxiety and increased RSA reactivity to the social cognition task.

### 2.4. Emotional Changes Assosciated with Challenging Behavior in Population with ASD

Many people with ASD have difficulty in communicating distress to family, carers and staff and this is a major impediment to managing emotional dysregulation and likely plays a role in some episodes of self-injurious behavior, as well as other challenging behaviors [37,38]. Challenging behavior, including self-injury, can be significant barrier to accessing community services, including education and can place unwanted restrictions on a person’s ability to participate in society. Additionally, challenging behavior has a profound impact on the emotional well-being of family, carers and staff [39]. 

As early as the mid-eighties, researchers were highlighting the potential benefit of supplementing the study of psychological and environmental variables with biological measurement [40] as a potential parameter in analyzing challenging behavior. A number of theories around the factors driving self-injury have suggested that it can be caused by a need to release tension, vent anger or modulate affect or by high levels of arousal [41,42]. The affect regulation model of self-injury [43] suggests that the behavior is driven by a need to control anger, anxiety or pain. Measuring biological parameters such as heart rate, pulse, and changes in temperature may well produce meaningful data to help predict these episodes. 

### 2.5. The Potential for Wearable Technology for Stress Detection in ASD in Daily Life

Our ability to measure biological variables has improved immensely in the last 10 years, which has culminated in discreet, socially acceptable devices, widely adopted by mainstream society. There is an increasing recognition that technology may play a critical role in improving our early detection of the onset of challenging behavior [37]. We now have the technology to examine in much more detail the role biological variables may have in anticipating and predicting episodes of self-injury and challenging behavior in people with ASD. Such devices could potentially be used to monitor physiological signals, which may correlate with internal emotional states, such as high levels of stress. Also, having a wearable device may help some individuals with ASD to increase their self-awareness of their internal emotional state and anxiety levels, so that they can follow certain behavioral techniques and coping strategies to help them self-regulate their emotions. This could be particularly useful for clients with comorbid alexithymia [44]. 

Researchers have been using various techniques including invasive tools for detecting stress/anxiety in individuals with ASD [45]. Conventional markers of stress are typically in blood, saliva, or urine. However, the tools associated with collecting those samples are either invasive or not suitable for real-time stress detection in daily use. The emergence of wearable assistive technologies provides a means for utilizing those technologies that can detect emotional arousal and corresponding changes in the autonomous nervous system non-invasively. Adopting such a wearable device can potentially carry enormous benefits for people with ASD and their caregivers. For example, such a device could potentially be used to monitor physiological signals which may correlate with internal emotional states, such as elevated level of stress. Monitoring the internal physiological and emotional states of the care-receiver may help them understand what they are experiencing in real-time. Furthermore, caregivers can take necessary actions to help the individual modulate their emotional state, for instance if the person is experiencing high levels of stress. 

## 3. Related Work

A number of reviews of relevant literature have been published, examining the technologies that can enable remote physiological and emotional monitoring, using various sensing modalities. For example, Majumder et al. [46] conducted a review on sensors used for remote monitoring for the general population. The authors compared various physiological and activity monitoring solutions aimed for the older population. Specifically, separate comparative studies for wearable monitoring devices of the cardiovascular system, body temperature, oxygen level parameters, and activity trackers were presented. Another work focused on wearable technology from a clinical perspective where Patel et al. reviewed wearable sensing technologies in several applications including wellness, safety, and home rehabilitation for older adults and individuals with chronic conditions [47]. 

Another category of reviews was aimed at works addressing systems that monitor physiological signals. For example, Kamišalic et al. [48] reviewed wristband wearable devices for physiological monitoring, activity monitoring, and environmental monitoring. However, although the review listed commercial devices as well as research prototypes, it was limited in its scope by focusing only on wrist-wearable devices and did not include other device form factors. Other reviews focused on certain types of sensors. For instance, Gao et al. reviewed wearable sweat sensors [49] whereas Li et al. reviewed flexible temperature sensors [50] and Nag et al. surveyed flexible physiological sensors [51]. 

Some researchers conducted review studies on wearable sensing technologies for a particular application. For example, Stacey et al. reviewed physiological systems for firefighters [47] and military personnel [52]. Baig et al. reviewed physiological devices with potential for clinical adoption [53]. Reinertsen et al. [54] reviewed physiological sensors for particular patients (for instance with neuropsychiatric illnesses). Iqbal et al. [55] reviewed wearable sensing technology in the medical domain and in hospital settings. 

Another group of researchers have reviewed devices and systems which claim to monitor emotional states of the user. For instance, Alberdia et al. [56] reviewed work conducted using automatic stress detection with physiological measurements and also psychological, and behavioral modalities, in addition to contextual measurement, which give more insights into the circumstances accompanying the stressful situation. Peake et al. [57] listed the features and evaluated the characteristics of a cross-section of wearable technologies for health and sports performance, and commented on which devices have been validated and which are reliable. 

More recently, the application of wearable technology to specific populations has been reviewed. People with certain conditions might have special needs and thus might require unique designs and functionality. Examples of such users include the ASD population. According to a survey conducted by Koo et al. [58], parents of individuals with ASD were particularly interested in being able to monitor their son or daughter’s physiological signals to understand anxiety levels and other emotions (72%). Cabibihan et al. [59] surveyed the research literature on different sensing technologies that can potentially be suitable for screening and intervention in ASD. Those sensing technologies were categorized into eye trackers, movement trackers, physiological activity monitors, tactile sensors, vocal prosody and speech detectors, and sleep quality assessment devices. The benefits and effectiveness of those devices in supporting the treatment of some symptoms of individuals with ASD were assessed, as well as their limitations. Although, the study was wide-ranging in its nature, covering a broad spectrum of sensing technologies used for early screening and treatment programs, the study was too broad to address the devices in detail, rather, it briefly highlighting the emotion assessing technology and focused on devices that use only one parameter. Taj-Eldin et al. [60] briefly reviewed commercial wearable devices for location tracking and emotion monitoring with particular focus on people with ASD. However, the study was limited in its scope as it partially covered physiological monitoring devices and the number of devices studied. 

As it can be seen, existing review papers either: study wearable devices for general or elderly population [46,47], focus on reviewing general physiological sensors, or the type of sensors such as flexible sensors [50,51], or physiological sensors and devices for certain workers (such as firefighters [47] or military personnel [52]), or on emotion assessment devices and algorithms targeted for the general publication, or they focus on the research prototypes designed for individuals with ASD [59]. However, to the authors’ best knowledge; little consideration has been given to reviewing commercial solutions or research prototypes that enable physiological and emotion monitoring with their potential application to ASD population. This paper focuses on reviewing devices and solutions that offer those functionalities, using a variety of physiological parameters.

## 4. Review of Physiological and Emotional Wearable Monitoring Solutions

In this section, we will explain the search methodology followed in conducting this study. Then, we will proceed to review commercially available physiological and emotion monitoring devices with various form factors and the associated sensing parameters. After that, we will review the state-of-the-art research solutions presented in the literature, showing their form factors, sensing capabilities, and their unique benefits. Then, emerging commercial solutions will be reviewed and discussed. Finally, a discussion on the overall physiological and emotion monitoring solutions will be provided. 

### 4.1. Research Methodology

The research methodology followed in conducting this review was as follows: The authors compiled a list of commercially available devices, emerging solutions, and research prototypes that collect and record any one or a combination of the physiological parameters discussed in Section 4.1. It should be noted that the search excluded fitness devices that only collect limited physiological parameters such as only heart rate (such as Fitbit Charge 3 [61]) unless it is accompanied by other physiological sensors (e.g., skin temperature, electrodermal activity, or electromyography sensor). The search was performed in the period starting from 1 January 2015 until the date of preparing this paper (i.e., October 2018). Devices that are included in the review were identified by searching the internet (e.g., Google Search Engine) for commercial devices. Research Prototypes that are included in the review were identified by searching the internet and databases of scientific literature (e.g., Google Scholar, PubMed) using the following keywords: “physiological wearable device”, “stress wearable device”, “emotional wearable device”, “emotional monitoring wearable device autism”, “stress wearable device autism”. Then, we broadly divided the technologies into the following three categories:

- Commercially available devices that can monitor physiological signals or emotional states.
- Research prototypes that can monitor physiological signals or emotional states.
- Forthcoming solutions that are still under development which can claim to be able to monitor physiological signals or emotional states.

Our review study investigates the key issues of: (a) what the wearable technology claims to do; (b) the parameters and sensors used; (c) the device form factor; (c) whether the technology has been independently validated against standard devices or validated devices; (d) its support for remote monitoring; (e) its suitability for people with ASD. Based on this information, we have evaluated a list of technologies and provided our own unbiased comments. It should be noted that the lists of products and research prototypes are not exhaustive. The compiled devices and prototypes are presented, evaluated and compared in the following sections.

### 4.2. Commercial Wearable Devices

The market is abundant with wearable devices that are aimed at physiological monitoring and, to a lesser extent, emotion monitoring. A list of commercially available devices is presented in Table 1. Those devices vary in the form factors, materials used, the sensors and parameters they use, their support for wireless connectivity and remote monitoring. Also, the target users for those devices are the general public and healthy people. However, those devices may be adopted by people with ASD. The autism spectrum is very broad, with some people with autism able to live fully independent lives and who may well be active consumers of such devices. At the other end of the spectrum are people with ASD and profound intellectual disabilities, who require high levels of support service, but who may also benefit from such devices being used by direct care staff to inform their intervention strategies and environmental management. More details on the potential clinical adoption of such technologies will be discussed in Section 6.

The devices presented Table 1 have various form factors including: wristbands and watches, (b) chest strap, vests, garments and shirts, patches, and sleeves. Each type of device has a certain size, materials, colors, and sensors that can collect certain physiological biomarkers. Examples of wristbands are the E4 Wristband and Embrace (Empatica, Inc., Cambridge, MA, USA), PulseOn OHR (PulseOn, Espoo, Finland), Apple Watch (Apple Inc., Cupertino, CA, USA). Examples of chest strap devices are BioHarness (Zypher, Inc., Dallas, TX, USA), EQ02 LifeMonitor (Equivital, Inc., Cambridge, UK), and Zepher belt (Medtronic, Inc., Minneapolis, MN, USA). Smart clothing has been increasingly popular, and it enables embedding the sensors within the cloth and can be a good alternative for the users who cannot tolerate intrusive devices such as wristbands. An example of such solutions is Hexoskin Smart Shirt, developed by Hexoskin Inc., Montreal, QC, Canada [62], which embeds fabric sensors that collect multiple parameters including ECG, heart rate, heart rate variability, respiration rate, and body movement data. An example of a sleeve device is the AIO Sleeve, developed by Komodo Technologies, Winnipeg, MB, Canada. According to the company, the device is capable of collect heart rate, heart rate variability using ECG fabric. However, there are no validation studies verifying the reliability of the data and if it can be used to make any clinical decisions.

An ever-increasing number of devices on the market can collect a single physiological parameter, such as heart rate. However, such devices when found, were excluded from the review as we were interested in devices that can collect multiple physiological signals with the potential for emotional monitoring. Furthermore, many of the devices with limited physiological sensing capabilities produce unreliable readings and cannot provide data of a clinical grade [63]. 

As can be seen in Table 1, some of the devices have limited functionality, including the collection of a single physiological parameter such as heart related data (Zypher-BioHarness, Inc., Annapolis, MD, USA and Polar H7-Polar Electro Oy, Kempele, Finland). Other devices collect, in addition to heart rate information, other physiological data including electrodermal activity (EDA), and Skin Temperature (ST). Examples of those devices are E4 Wristband (Empatica, Inc.), Visi Mobile (Sotera Wilress, Inc., San Diego, CA, USA), EQ02 LifeMonitor (Equivital, Inc., Cambridge, UK). Other products have the capability of collecting information about the respiratory system, such as ViSi Mobile (Sotera Wilress, Inc.), QardioCore (Qardio, Inc., San Francisco, CA, USA). The majority of these devices include sensors for daily physical activity and posture recognition. More recently, new physiological monitoring devices provide metrics for the assessment of sleep quality. 

Examples of these products are Vivosmart 4 (Garmin, Inc.), GoBe2 (Healbe, Corp.), BioStamp nPoint (MC10, Inc.), and AO Sleeve (Komodo Technologies, Inc.).

It should be noted that very few of these devices are certified by regulatory bodies as medical devices (such as E4 Wristband and Embrace) with the majority being consumer electronics products for health and fitness applications. The validity of the devices varies as some have been clinically tested on healthy volunteers. Even among the devices with some validation data, few have been validated and evaluated in terms of their usefulness to consumers. Therefore, the authors have conducted a search and included validation studies, when found, that are peer-reviewed or alternatively have followed some scientific methodology. Few of those studies validated the device against gold standards, but rather against other reliable physiological monitors. For instance, Apple Watch has been validated against Polar H7 chest strap where RR interval series have shown good reliability (i.e., Lin’s concordance correlation coefficient > 0.9 and intraclass correlation coefficient > 0.9) and agreement coefficient (i.e., agreement = (1-information-based measure of disagreement) > 0.9) [96]. Also, the HRV parameters derived from RR interval series were compared and their ability to identify autonomic nervous system (ANS) response to mild cognitive stress and the results showed very good reliability and agreement (>0.9) [96]. BioHarness 3.0 has been evaluated for heart rate measurements and was demonstrated to be reliable for mean heart rate data but not for heart rate variability analysis [75]. EQ02 LifeMonitor has been validated against a gold standard Holter ECG monitor and showed variable Pearson correlation coefficient ranging from 0.724 to 0.997 depending on the artifacts content in the signal. The accuracy and precision of the EQ02 Life Monitor is highly dependent on artifact content. Suunto Smart Belt has been also tested on healthy volunteers and compared against gold standard ECG monitor during an orthostatic tilt test [94]. The data indicated excellent accuracy for the device, with minimal error rates and strong HRV data correlation with that of the reference device.

Some of the listed devices are more suited to people with ASD or intellectual disability (ID) than others. The suitability of each device was assessed using a combination of criteria. The assessors partially used the findings of a survey conducted by Koo et al. who interviewed people with ASD and their parents. The suitability index in our study ranges from one to five points/stars in Table 1 where one star means not very suitable for people with ASD/ID and five stars indicates highly suitable device for ASD/ID population. Koo et al. [58] concluded that, in general, accessories like wristbands and watches are preferred by people with ASD and thus are given higher scores (three to five stars) in our review. This finding is in agreement with results of interviews conducted by Notenboom when asking children with autism about their preferred device type, where 70% reported that the watch was the most preferred choice followed by patches with a preference rate of 30% [104]. On the other hand, Koo reported that garments and shirts are the second preferred device. The discrepancy between the two studies may be due to the difference in age between the samples: Koo et al. interviewed adults with ASD whereas Notendoom interviewed children. In addition, Notendoom offered a limited number of potential device form factors and did not include vests or shirts in the device list.

Therefore, in our review, for devices including garments, vests, and sleeves, they can receive medium to high score on the Suitability Index. Obtrusive devices such as chest belts are the least suitable for people with ASD/ID. 

### 4.3. Research Prototypes

Several prototype devices for emotion monitoring have been developed and are reported in the literature, that can be used for emotion recognition and stress management applications. For example, Imani et al. have developed a hybrid wearable sensor that includes both chemical and physical sensors in one patch. The patch can be used to monitor physical exertion, and electrocardiogram measurements can be used to monitor heart health and function [105]. The sweat lactate sensor can be used to track an individual’s performance and exertion level. The hybrid device has the potential to be programmed to recognize strong emotional states such as stress and anger, once suitable algorithms have been developed. 

Another category of devices focuses on utilizing multiple physiological parameters for stress detection. For instance, Yoon et al. have developed wearable flexible patch for stress monitoring [106]. The system detects three bio-signals namely; skin conductance, skin temperature, and arterial pulse wave, simultaneously utilizing a multi-layer sensor resulting in small contact area (25 mm×15 mm) and flexible material which improves wearability. 

The process of developing a physiological or emotion monitoring device should take many factors into account. Some of those factors include the user needs and tolerance, device form factor, and the flexibility of the device, to name a few. Further, when the target users are people with ASD, additional stringent design requirements are imposed [58]. For example, many people with ASD can tolerate only flexible devices made of soft materials as they have hyper-sensory issues. This implies that the device should be embedded into their clothing or used as part of the mainstream technology (e.g., wristbands and smart watches). 

There are several research prototypes that have been developed where some are designed specifically for people with ASD, or some are developed for general population with a potential application to ASD population. An example of such a device is a scarf-based emotion state monitor which has been developed by Williams et al. [107]. The authors claim that the proposed device aims to eventually reflect on the user’s emotional state, modify his/her affect, and interpret the emotional states of others. A universal design was utilized that includes people with disabilities and the chosen scarf form factor was perceived to reduce stigma associated with medical devices, by making it as an accessible technology with a fashionable design. According to the authors, the system, upon completion, would recognize five psycho-physiological constructs (also called affective states), namely stress, sad, calm, happy, and excited and include a feedback control system for intervention that responds to certain emotional states (e.g., playing upbeat music when the user feels sad) to mitigate negative emotions and promote positive ones in real-time using actuations. However, this paper presented only the physiological measurement device and did not develop the algorithms or methods that will assess such affective states, nor did it address the clinical utility of such interventions as playing music during episodes of stress. Another smart scarf has been developed by Guo et al. where the device uses a heart rate sensor and a Electrodermal Activity (EDA) sensor to detect and recognize emotional information [108]. According to the authors, this scarf can also respond to negative emotions when detected, by changing its color and emitting an odor to promote positive emotions. However, this paper presented only the physiological measurement device and did not develop the algorithms or methods that can recognize relevant affective emotional states.

Some other researchers have investigated other device types. For example, Hui et al. also developed a smart glove that utilizes different physiological sensors [109]. This device used a photoplethysmograph (PPG) sensor for heart rate, electrodermal activity, skin temperature, and electromyography (EMG) sensors. However, the EMG signal showed no significant relationship between signals on different parts of the hand and thus would not be reliable for emotion state recognition. The system was programmed to recognize five emotions (happiness, anger, fear, disgust, sadness) and was tested on sample healthy volunteers who viewing short affective video films. This paper focused only on the preprocessing of emotion recognition, particularly on the analysis of the validation of successful affective moments stimulated by audiovisual cues through simple feature comparison. Therefore, it did not develop the algorithms that make use of those features necessary for emotion recognition. 

Another drawback of this study is that it was only tested on healthy participants and did not include people with disabilities such as ASD. A smart glove that is designed for ASD population was developed by Koo et al. [58]. The glove included an EDA sensor and pulse oximeter sensor. The EDA sensor was made of conductive thread sewn into the glove to make electrical contact with the skin. The pulse oximeter sensor was used to measure heart rate and heart rate variability through a PPG. The glove incorporated a wireless module enabling a remote monitoring and notification for individuals with ASD and their parents. 

Other researchers investigated the potential of wearable technology to enable people in the general population to recognize the emotions of people with ASD. For instance, Tang et al. used a novel approach that utilizes multiple sensing modalities, namely physiological data (HR, perspiration); behavioral meters for body gestures and motions; and facial expressions [110]. Although the platform provides a good preliminary functionality, the system was only able to distinguish between two basic emotion (i.e., sadness and happiness). 

The aforementioned research prototypes and other state of the art devices have been compiled in Table 2. The authors comment on their suitability for use for people with ASD by providing a score on a scale from one to five stars. Also, when the device is specifically designed for people with ASD, it receives high score (i.e., four or five stars on the five-point rating scheme).

### 4.4. Emerging Solutions

More recently, solutions have been under development that utilize advanced artificial intelligence technologies such as machine learning and deep learning for emotion recognition to make meaningful information out of the collected physiological data. For example, MyFeel wristband, from Sentio Solutions Inc [115], uses proprietary algorithms to process the data where it collects heart rate, electrodermal activity and skin temperature. The device includes a mobile app aimed at coaching and stress management using cognitive-behavioral techniques.

Another device that is still under development and targeted for people with ASD is called Reveal, from Awake Labs [116]. This device collects heart rate, electrodermal activity and skin temperature data. The solution integrates a patented technology called Anxiety Meter to assess anxiety level of the individual in a natural setting and can notify the caregiver when anxiety levels start to elevate by applying data analytics techniques to make “smart” clinical decisions [117]. Gaia Wearable from GAIA LLC., Louisville, CO, USA [118] uses a shirt with embedded sensors to monitor signals of stress and notifies individuals with ASD or their caregivers of “behavioral meltdowns”. mPath is a startup company that produces smart sensor called MOXO Wearable that uses skin conductance to monitor the emotional state of the user and detect stress. The company developed an algorithm based on data collected during clinical trials monitoring electrodermal activity to predict emotions [119]. 

Another class of solutions aim to do more than monitor stress level but also manage and reduce stress utilizing innovative engineering and neuroscience concepts. An example of such technologies is the TouchPoints wristband produced by Touchpoint Solution™, Scottsdale, AZ, USA [120]. This solution provides emotional monitoring but also claims to relieve stress using stimulating electrical pulses that a patented technology named Bi-lateral alternating stimulation–tactile (BLAST) [121]. Serin et al. have reported a significant reduction in the levels of both emotional stress and bodily distress (i.e., 62% and 50%, respectively) after applying BLAST technology suggesting its potential benefits on the stress response by reducing both emotional stress and disturbing body sensations, though their study lacked blinding and could be subject to a placebo effect. Bio Hug Technologies Ltd., Kiryat Tiv’on, Israel [122] designed a vest that uses air pressure to calm people with extreme levels of stress such as people with Attention deficit hyperactivity disorder (ADHD), post-traumatic stress disorders (PTSD), and ASD.

Recently developed stress monitor solutions have been both physiological data and other additional information. Muaremi et al. [123], Gjoreski et al. [124], and Padmaja et al. [125] have included contextual information such as voice, physical activity, and sleep data for comprehensive assessment of stress and the associated activities. 

A new generation of stress management and stress relief devices is emerging using state of the art innovations in electrical and biomedical engineering. For instance, Thync developed by Thync Global Inc., Los Gatos, CA, USA [126] uses two neuro-stimulation technologies, namely Transcutaneous Electrical Nerve Stimulation (TENS) and Transcranial Direct Current Stimulation (tDCS). When using TENS technology, small electrical impulses are delivered to certain areas of the body, where the signals are transmitted through the spinal cord and reach brain, which may help alleviate stress levels by relaxing the muscles. This technology is currently being validated, with a pre-clinical study showning the potential of TENS for stress reduction [127]. tDCS, on the other hand, applies a small electrical current, that lead to the polarization of the neural tissue and modulation of neural activity and has shown its potential for preventing chronic stress in a preliminary animal study [128]. As can be seen, some technologies are in their early stage of testing and not thoroughly validated and will require extensive validation to show their usefulness in clinical studies. Such technologies which have been in use in other applications such as pain relief, could be applied in stress relief applications. 

Recent developments in neuroscience have allowed the use of EEG signals for emotional state identification. An example of a device that applies this technology is Muse produced by InteraXon, Toronto, ON, Canada [129]. The device guides the user’s meditation through changing sounds of weather based on the state of the brain. The developers claim that it creates a deeper sense of focus and motivation. As shown in Section 2.2.6 stress may cause a rise in blood pressure among other physiological indicators of stress. A start-up company has recently developed wristband prototype produced by Omron Healthcare [130]. The device measures blood pressure and heart rate reliably using optical sensors which allow the possibility for developing stress monitor and detection solution. 

New trends in technological developments will stimulate further developments in stress monitors. For example, developers are working on a system that can proactively predict stress using measurements of physiological data, activity level, and sleep quality. Leaf Urban wristband from Leaf Urban, Inc., San Francisco, CA, USA [92] claims that using propriety algorithms, the system can warn the user of un-healthy physical activities and decreased sleep quality that is likely to lead to increased stress levels, based on data from current readings and sleep report of the night before.

The aforementioned solutions are presented and compared in Table 3. Also, the suitability and potential adoption by people with ASD is assessed. Overall, there is an ever expanding market of wearable solutions that use innovative and state of the art technologies bringing together many advances in diverse fields such as neuroscience, electrical and electronic engineering, psychology, psychiatry, and education. This rapid growth is expected to continue in the years to come.

## 5. Discussion

**Figure 1:**
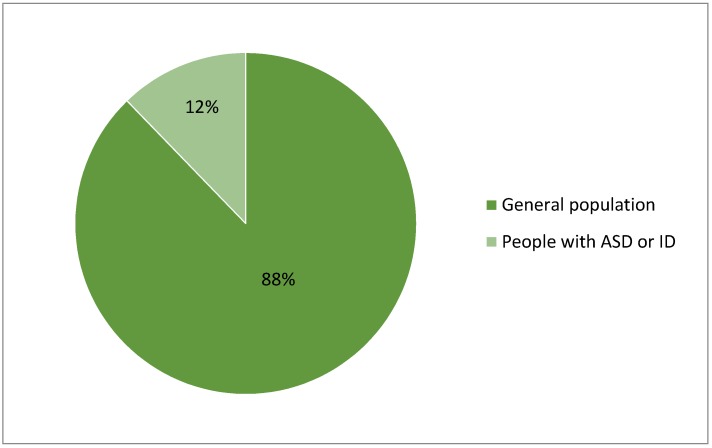
Proportion of products designed for general population vs. people with ASD.

In the previous section, we reviewed an extensive list of products and solutions used for physiological monitoring or emotional state recognition application. As seen in Section 4, the majority of the reviewed devices are designed for the general population. As can be seen in Figure 1, the proportion of products that are specifically designed for people with ASD is very small. While this seems disappointing at first glance, some physiological monitoring devices can still be adopted for use in people with ASD. The devices can be divided into two categories; the first focus purely on physiological measurement, while others are aimed at analyzing the physiological signals, with the potential to make algorithmically-based interpretations of the person’s emotional state. By building on the available devices for physiological measurements, specific applications for ASD may then focus on the analysis methods, without having to again validate the physiological measurement methods. Best of breed solutions may be combined, as needed. Also, some of the physiological measurement devices reviewed in this paper can be useful for multiple data analysis such as stress detection, quality of sleep assessment, and physical activity classification. Examples of those devices include: GoBe2 from Healbe, Corp., Biostamp nPoint from MC10, Inc., and AIO Sleeve from Komodo Technologies, Inc.

Another finding of this review is that although there are several research efforts driven mainly by academic institutions to develop wearable emotion monitoring technologies for people with ASD, a small number of wearable devices can make their way to market mainly due to the relatively small market size of people with ASD. This challenge can be addressed by forming a consortium of stakeholders including health service management, service providers, researchers, product designers, clinicians, engineers, entrepreneurs, and venture capitals to develop a strategy and business model that can ensure a suitable market size and attainable profits while adequately providing wearable assistive technology for the under-represented users.

When parents and caregivers assess the potential of using assistive wearable technology for emotional assessment, there needs to follow a multi-element approach. First, they need to consider a suitable device type for the particular user. Users, caregivers or service providers should identify the intended purpose of utilizing such technology and how it may complement their current intervention and stress management programs. 

Then, parents and caregivers will need to take into account important attributes for individuals with ASD who will ultimately decide if they will adopt the wearable device. Therefore, caregivers and service providers need to carefully consider those attributes for individuals with ASD when choosing and using an assistive wearable technology. These include the durability of the device, but more importantly the comfort of the device, which is often reported as the most important factor [58]. Almost half of individuals with ASD and their parents prefer to use designs that are unlikely to be noticed and this is crucial for long-term monitoring. The device should be as similar to mainstream products as possible to mitigate the risk of associated stigma. They also reported a preference for devices that are made of flexible materials and are soft and are easy to wear and remove when needed.

About half of the surveyed individuals prefer to use wearable technology which can monitor body signals such as heartbeat and respiration and those are related to difficulties with monitoring or being aware of their own emotional state. More interestingly, they asked for devices that give them alerts when signs of negative emotions are detected (e.g., the user is stressed).

According to the survey of Koo et al. [58], parents or caregivers of individuals with ASD report that they prefer functions related to wearable technology that aid awareness and management. For instance, parents would like to be able to monitor the vital body signals of care receivers to understand their emotions and anxiety level so they can help them to relieve stress. Specifically, they reported that one of the most common challenges for individuals with ASD is regulating their own emotions and managing their anxiety and having control over sources of stress. Based on those findings, the type of wearable technology and device form factor should be carefully selected. 

**Figure 2:**
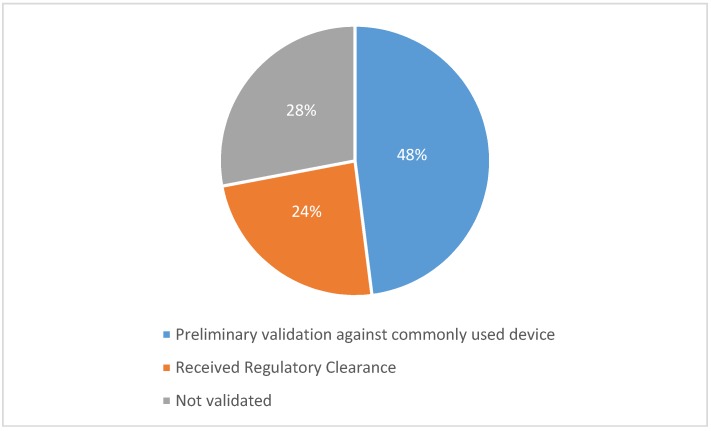
The chart shows the proportion of reviewed commercial devices (in Section 4.2) and their type of validation, if validated.

Another important aspect when selecting an appropriate wearable assistive device is to ensure the validity and effectiveness of the device for its intended use. This review has revealed that for physiological monitoring devices seen in Section 4.2, there is almost an absence of scientifically validated data against gold standards for wearable physiological measurement. However, researchers often use cross validation against another commonly used device which has widely accepted effectiveness and good reliability. Figure 2 shows that almost half of the commercially available devices have used commonly-used devices for testing and showing preliminary validity on a small number of people. Another quarter of the reviewed devices have received regulatory clearance (e.g., FDA Clearance) which does not prove its clinical validity, though. The rest of the products have not proven their clinical validity through scientific peer-reviewed studies. 

As seen in Section 2.3, people with ASD may have different physiological response to stressors. Therefore, in addition to the unique device design requirements, specific algorithms and data analytics tools should be developed to serve people with ASD.

## 6. Conclusions

Wearable physiological and emotion monitoring devices are becoming popular and are able to perform multiple functions. In addition to the usefulness of those devices for the general population, they have the potential to help people with ASD, their parents, and caregivers. The main aim of this work was to give a comprehensive overview of the research and to report the range of solutions in the area of physiological and emotion monitoring and recognition. In this review paper, we have reported commercial products, emerging solutions as well as research works to evaluate, compare and analyze the functions they can perform, the sensors and parameters they collect. Also, we have commented on the suitability of those devices for people with ASD. In preparing this paper, we studied the literature extensively from various aspects. Based on consultations with clinicians including psychologists, we believe that the key parameters in any device are the heart rate and electrodermal activity. Therefore, potential developers of related application should have those sensors at a minimum. We also analyzed relevant studies in which individuals with ASD were interviewed and their preferences for the wearable technology were documented. In summary, the target device should small, flexible, unobtrusive, and discreet in design. Examples of potential devices are watches and smart clothing (such as vest). There are challenges for the potential clinical adoption of those wearable technologies. First, most existing devices on the market are designed for the general population with little attention to people with ASD, though this appears to be changing. Second, the use of these devices requires training for users and their caregivers. Third, even when devices are designed for people for ASD, the cost of purchasing and gaining continuous service for some devices can be expensive for parents and caregivers. Therefore, support from the health service and disability sector is important. Collaboration between health services and business, such as entrepreneurs, venture capitals, and caregivers, to manage this cost, will help in taking the first steps to the adoption of this technology and ultimately reduce the economic burden on carers.

## Figures and Tables

**Table 1:** Comparison of commercial physiological and emotional monitoring devices.

Product, Producer	Purpose	Device Form Factor	Sensors/Parameters	Support for Wireless Technology	Clinical Validity	Suitability for Individual with ASD
Vivosmart 4, Garmin Inc., Olathe, KS, USA [64]	Monitoring stress score using HRV, sleep quality, and activity (step, distance, calories, floors)	Wristband	HR, HRV, accelerometer, oxygen saturation	Yes, Bluetooth Low Energy (BLE) and ANT+	Not clinically validated	☆☆☆
Rhythm24, Scosche Inc., Oxnard, CA, USA [65]	Monitoring heart rate data	Armband	HR, HRV	Yes, BLE/ANT+ Connection	Investigation showed reliable readings [66]	☆☆☆
E4 wristband, Empatica Inc., Cambridge, MA, USA [67]	Collecting physiological and movement data only	Wristband	HR, HRV, EDA, ST, acceleration	Yes, BLE and data uploaded to cloud	Comparable readings to chest strap [66]	☆☆☆☆
Embrace, Empatica Inc., Cambridge, MA, USA [68]	Alerts epileptics (or caregivers) when an attack occurs in ASD population	Watch	HR using PPG, EDA, ST, and activity	Yes, using BLE	Clinically validated and FDA approved [69]	☆☆☆☆☆
PulseOn OHR, PulseOn, Espoo, Finland [70]	Monitoring physiological and movement data	Wristband	HR, HRV, acceleration	Yes, real-time transfer to application using BLE	HR/HRV are reliable only during resting condition [71]	☆☆☆
ViSi Mobile, Sotera Wireless Inc., San Diego, CA [72]	Monitoring physiological data	Wristband	ECG, HR, pulse rate, BP, RR, SpO2, and ST	Yes, using WiFi	FDA approved for blood pressure monitoring [73]	☆☆☆
BioHarness 3.0, Zephyr Technology, Annapolis, MD, USA [74]	Physiological and activity data collection	Chest strap	HR, HRV, EDA, body temperature, RR, activity, location	Yes, using BLE and LP–PAN [74]	Only reliable HR but HRV is not suitable for clinical evaluations [75]	☆☆
EQ02 LifeMonitor, Equivital Inc., Cambridge, UK [76]	Physiological and activity data collection	Chest Belt	ECG; HR, HRV, RR, EDA, ST, acceleration	Yes, using Bluetooth 2.1	EQ02 can accurately measure ECG and HRV [77]	☆
Zephyr belt, Medtronic, Inc., Minneapolis, MN, USA [78]	Sports health monitoring	Belt	HR, HRV, RR	Yes, using BLE	Not clinically validated	☆
Hexoskin Smart Shirt, Hexoskin, Inc., Montreal, QC, Canada [62]	Physiological and activity data collection and monitoring quality of sleep	Shirt	HR, HRV, Heart rate recovery, RR and volume, acceleration and power	Yes, using BLE and uploaded to cloud	Clinically validated to obtain reliable HR and RR [79]	☆☆☆
Polar H7 & H10, Polar Electro Oy, Kempele, Finland [80]	Monitoring HR activity	Chest Strap	HR, HRV using ECG	Yes, BLE protocol	Clinically comparable data [81,82]	☆
Polar V800, Polar Electro Oy, Kempele, Finland [83]	Monitoring HR activity	Watch	HR, HRV using light source sensor	Yes, BLE protocol	Clinically validated during rest [84]	☆☆☆☆
Polar Vantage V, Polar Electro Oy, Kempele, Finland [85]	Monitoring HR and physical activity	Watch	HR, HRV, accelerometer	Yes, BLE protocol	Not clinically validated	☆☆☆☆
QardioCore, Qardio, Inc., San Francisco, CA, USA [86]	Monitoring HR, ST, activity tracking	Can be mounted on chest	HR, HRV using ECG sensor, RR, ST, acceleration	Yes, BLE protocol	FDA (510) cleared [87]	☆
Armour39^®^ Module & Chest Strap, Under Armour, Inc., Baltimore, MD, USA [88]	Monitoring heart rate, calorie	Chest Strap	HR, HRV using ECG	Yes, BLE protocol	HR/HRV are reported to be accurate [89]	☆
Wahoo TICKR X, Wahoo Fitness, Atlanta, GA, USA [90]	Monitoring HR, calorie tracking, insights of physical activity	Chest Strap	HR, HRV using ECG acceleration	Yes, using BLE and ANT+	Bluetooth is HR/HRV accurate, ANT+ is just HR accurate [91]	☆
Suunto 9, Suunto, Inc., Vantaa, Finland [92]	Monitoring HR activity	Watch	HR, HRV	Yes, using BLE	Not clinically validated	☆☆☆☆
Suunto Smart Sensor, Suunto, Inc., Vantaa, Finland [93]	Monitoring HR activity	Chest Strap	HR, HRV	Yes, using BLE	Tests showed reliable HR and HRV [94]	☆☆
Apple Watch Series 4, Apple, Inc. [95]	HR and physical activity	Watch	HR, HRV	Yes, using BLE and to cloud	FDA cleared, initial tests showed good HR and HRV data [96]	☆☆☆
GoBe 2, Healbe, Corp., Moscow, Russia [97]	Emotional monitoring, calorie intake, sleep quality	Wristband	HR, EDA, impedance, BP, and acceleration sensor	Yes, using BLE	Not clinically validated	☆☆☆
BioStamp nPoint, MC10, Inc., Cambridge, CA, USA [98]	Monitoring heart rate, activity and posture data, sleep quality	Patch	HR, HRV, respiration rate, acceleration	Yes, using BLE and uploaded to cloud	FDA (510) cleared [99]	☆
VitalPatch, VitalConnect, San Jose, CA, USA [100]	HR, HRV, respiration rate, skin temperature, body posture, and activity	Patch	ECG for HR acceleration, thermistor for ST	Yes, data uploaded to the cloud	FDA (510) cleared, CE Marked, ISO 13485 certified.	☆
Lief Smart Patch, Lief Therapeutics, Inc., San Francisco, CA, USA [101]	Monitoring heart rate, emotional state, activity	Patch	HR, HRV using ECG sensor, RR, acceleration	Yes, using BLE	Not clinically validated	☆☆☆
AIO Sleeve, Komodo Technologies, Inc., Winnipeg, MB, Canada [102]	Physiological, activity data, monitoring stress level and sleep quality	Sleeve	HR and HRV using ECG, acceleration	Yes, using BLE protocol	Not clinically validated	☆☆☆☆
Empower Me, Brain Power, LLC., Cambridge, MA, USA [103]	Recognizing others’ emotions for ASD people	Glass	Acceleration	Yes, using BLE protocol	Clinically validated	☆☆☆☆

**Table 2:** Comparison of physiological and emotional recognition/monitoring research prototypes.

Reference, Institution, Year	Purpose	Device Form Factor	Sensors/Parameters	Support for Wireless Technology	Clinical Validity	Suitability for ASD
Imani et al. [105], University of California, San Diego, 2016	Heart rate activity and sweat monitoring	Patch	HR from ECG sensor, concentrations of hydrogen and sodium ions, lactate in human sweat	Yes, using BLE protocol	Initial test on three subject and HR sensor comparable to commercial monitors	☆☆
Yoon et al. [106], Advanced Institute of Science and Technology, 2016	Stress Monitoring	Patch	EDA, ST, and pulsewave	No	No	☆☆☆
Williams et al. [107], Microsoft, Inc., Redmond, WA, USA 2015	Reflecting on user’ emotional state, recognizing other’s emotions and stress-relief system	Scarf	Heart rate (HR)	Yes, using BLE	Tested on small number of participants but not validated yet	☆☆☆☆☆
Guo et al. [108], Purdue University, 2016	Recognizing emotions, enhancing positive emotions and mitigating negative ones.	Scarf	HR, Electrodermal activity (EDA), and use color-changing and odor emitting tool to promote positive emotions	Yes, using Infrared technology	Not validated yet	☆☆☆☆☆
Tang et al. [110], Wenzhou-Kean University, 2016	Helping neuro-typical individuals recording emotional states of children with ASD		HR, perspiration, Pressure, and acceleration sensor	Yes, using WiFi to upload data to cloud	No	☆☆
Parlak et al. [16], Stanford University, 2018	Measuring stress noninvasively	Patch	Cortisol using novel electrochemical sensor	No	Not validated yet	☆☆☆☆
Notenboom [104], University of Twente, 2017	Monitoring stress level for people with ASD	Watch	HR, EDA, ST, and oxygen sensor	No	Not validated yet	☆☆☆☆☆
Torrado et al. [111], Autonomous University of Madrid, 2017	Inferring outburst patterns and self-regulation	Watch	HR monitor, accelerometer and gyroscope	Yes, BLE protocol	Not validated yet	☆☆☆☆
AlHanai et al. [112], Massachusetts Institute of Technology, 2017	Predicting the mood using para-linguistic cues, linguistic content and the physiological state	Watch	ECG, PPG, accelerometer, gyroscope, bioimpedence, electric tissue impedance, EDA, ST, audio and text features	Yes, BLE protocol	Not validated yet	☆☆☆☆☆
Northrup et al., [113], The Center for Discovery, USA, 2016	Monitoring stress to coordinate emotional arousal with contexts in ASD population	wristband	Electrodermal Activity (EDA)	Yes, BLE protocol	Preliminary tests showed good results [113]	☆☆☆☆
Simm et al. [114], Lancaster University, 2017	Assessing anxiety	Wristband	HR, ST, and environmental sensing	No	Preliminary tests on adults with ASD [114]	☆☆☆☆☆
Hui et al. [109], University of Reading, 2018	Emotional monitoring and assessment	Glove	Blood volume pulse, EDA, ST	Yes, using BLE to phone and data uploaded to cloud using WiFi/Cellular	Not validated	☆☆☆☆
Koo et al. [58], Konkuk University & University of California, Davis, 2018	Monitoring Emotional states for people with ASD	Glove	EDA, HR and HRV using pulse oximeter	Yes, using BLE protocol	Not validated	☆☆☆

**Table 3:** Comparison of emerging solutions for emotional monitoring.

Solution Name, Company	Purpose	Device Form Factor	Sensors/Parameters	Support for Wireless Technology	Clinical Validity	Suitability for Individuals with ASD
MyFeel wristband, Sentio Solutions Inc., Palo Alto, CA, USA [131]	Recognizing emotions	Wristband	HR, EDA, ST	Yes, using BLE	Preliminary study showed usefulness on 150 subjects	☆☆☆☆
Reveal, Awake Labs, Toronto, ON, Canada [34]	Monitoring stress and anxiety in ASD	Wristband	HR, EDA, ST	Yes, using BLE	Undergoing clinical validation and pilot study [132]	☆☆☆☆☆
Gaia Wearable, Gaia LLC., Las Vegas, NV, USA [118]	Monitoring stress, notifying individuals with ASD of meltdowns	Shirt	HR, EDA	Yes, using BLE	Not validated	☆☆☆☆☆
Dymaxia Wearable, Dymaxia, Inc., Toronto, ON, Canada [133]	Analyzing anxiety trends in ASD	Wristband	HR	Yes, using BLE	Not validated	☆☆☆
BioHug Vest, Bio Hug Technologies Ltd., [122]	Calming people with extreme levels of stress in people with ADHD, post-traumatic stress disorders (PTSD), ASD	Vest	Air pressure using Deep Pressure patent technology	Yes, using BLE	Not validated	☆☆☆☆☆
MOXO Wearable, mPath, Broomfield, CO, USA [134]	Monitoring stress levels	Watch	Electrodermal activity	No known	Preliminary studies showed good results [119]	☆
iBreve, iBreve, Ltd., Dublin, Ireland [135]	Monitoring breathing, stress, and activity for women	Clip	RR, HRV, and acceleration	Yes, using BLE	Initial tests to distinguish between different breathing [136]	☆☆☆
KiddoWear band, Good Parents Inc., San Francisco, CA, USA [137]	Monitoring health, stress, activity, and sleep levels	Wristband for kids	HR, ST, EDA, and acceleration	Yes, using BLE	Not validated yet	☆☆☆☆
Spire Health Tag, Spire, St. Louis, MO, USA [138]	Monitoring respiration and HR, sleep quality	Tag	HR, RR, and acceleration	Yes, using BLE	Undergoing clinical trial [139]	☆☆☆☆☆
SKIIN Smart Clothing, Myant Inc., Toronto, ON, Canada [140]	Monitoring respiration heart rate, and sleep	Textile clothing	HR, ST, RR and acceleration	Yes, using BLE	Not validated yet	☆☆☆☆☆
U-Check-It, Bedell Innovations, LLC., PA, USA [141]	Monitoring stress level, stress relief	Wristband	EDA, body temperature, acceleration	Yes, using BLE	Not validated yet	☆☆☆☆
Moodmetric Smart Ring, Vigofere Oy, Tampere, Finland [142]	Monitoring stress level	Ring	Electrodermal activity	Yes, using BLE	Not validated yet	☆☆☆☆
TouchPoints wristband, Touchpoint Solution™, [120]	Stress and anxiety relief	Wristband	Bi-lateral alternating stimulation –tactile (BLAST)	No	Patent-pending neuro-scientific technology	☆☆☆☆
The Pip, Galvanic, Inc., Calgary, AB, Canada [143]	Monitoring and reducing stress level	Teardrop-shape	Electrodermal activity	Yes, using BLE	Not validated yet	☆
Thync, Thync Global Inc., USA [126]	Improving mood (reduce stress, relax, and help in sleep)	Wearable on forehead	Neurostimulation (TENS and tDCS)	Yes, using BLE	Not validated yet	☆
Muse, InteraXon [129]	Monitoring brain activity and stress-relief	Headband	EEG sensor	Yes, using BLE	Preliminary tests [144] and undergoing clinical trial [139]	☆
HeartGuide, Omron Healthcare, Milton Keynes, UK [130]	Monitoring blood pressure, heart rate, sleep quality	Wristband	Optical blood pressure sensor	Yes, using BLE	Undergoing clinical validation but not validated yet.	☆☆☆☆
Leaf Urban, Bellabeat, San Francisco, CA, USA [145]	Stress prediction, sleep monitoring	Bracelet, necklace	Monitor activity, sleep, and RR	Yes, using BLE	Not validated yet	☆☆☆

## Abbreviations

The following abbreviations are used in this manuscript:

BLE	Bluetooth Low-Energy
HR	Heart Rate
HRV	Heart Rate Variability
RR	Respiration Rate
RSA	Respiratory Sinus Arrhythmia
ST	Skin Temperature
EDA	Electrodermal Activity
PPG	Photoplethysmogramy
BVP	Blood Volume Pulse
ECG	Electrocardiogramy
EEG	Electroencephalogramy
EMG	Electromyography
FDA	Drug and Food Administration
TENS	Transcutaneous electrical nerve stimulation
tDCS	Transcranial direct current stimulation
